# Effects of crocin on the enhancement of *in vitro* neurogenesis: Involvement of Notch and CREB/BDNF signaling pathways

**DOI:** 10.22038/IJBMS.2024.76308.16513

**Published:** 2024

**Authors:** Shayan Vafaei, Vida Mirzaie, Masoumeh Baghalishahi, Elahe Mousanejad, Seyed Noureddin Nematollahi-mahani

**Affiliations:** 1 Department of Anatomical Science, Afzalipour School of Medicine, Kerman University of Medical Sciences, Kerman, Iran; 2 Kerman Neuroscience Research Center (KNRC), Institute of Neuropharmacology, Kerman University of Medical Sciences, Kerman, Iran; 3 Afzal Research Institute (NGO), Kerman, Iran

**Keywords:** ADSCs, CREB/BDNF, Crocin, Neurogenesis, Notch

## Abstract

**Objective(s):**

Adult neurogenesis, the process of generating new neurons, continues throughout life. Unfortunately, this process is insufficient in pathological conditions and needs to be promoted. Crocin, the active component of saffron, affects neurogenesis* in vivo* and *in vitro*. We aimed to investigate the enhancing effects of crocin on the neurogenesis of adipose-derived mesenchymal stem cells in the presence of retinoic acid, as well as the molecular pathways involved.

**Materials and Methods:**

Differentiation capacities and stemness potential of harvested ADSCs were evaluated by differentiating into osteocytes and adipocytes, and expression of mesenchymal CD markers by flow cytometry. The optimum dose of crocin was assessed with an MTT assay. Crocin, retinoic acid, CREB/BDNF, and Notch inhibitors and their combination were added to the culture medium. Jag1, Hes1, Notch, and BDNF gene expression were analyzed by RT-PCR on days 7, 14, and 21, while CREB, DCX, SOX2, and NeuN expression were analyzed by immunofluorescence.

**Results:**

Expression of mesenchymal CD markers as well as adipogenic and osteogenic differentiation confirmed the origin and properties of ADSCs. The optimal dose of crocin was 1 mM. Crocin significantly (*P*<0.05) increased, while inhibitors (DATP&Naphthol) significantly (*P*<0.05) decreased Jag1, Hes1, Notch, and BDNF expression. Immunofluorescent assessments showed that expression of DCX, BDNF, NeuN, and Sox2 proteins increased significantly (*P*<0.05) after crocin administration and decreased significantly (*P*<0.05) after inhibitor administration.

**Conclusion:**

Crocin can be used as an enhancer for neural differentiation of MSCs* in vitro* in the presence of retinoic acid. The mechanism is proposed through Notch and CREB/BDNF signaling pathways.

## Introduction

Adult neurogenesis, an important and novel finding in the field of neurology, is the process of generating new neurons in the adult CNS (Central Nervous System) (1, 2). Despite what was once believed, it is now known that neurogenesis continues throughout the life of *Homo sapiens* (1, 3, 4). Neural stem cells (NSCs) are capable of proliferating and differentiating into different neural cell lines such as neuroblasts and glioblasts (5, 6). Neurogenesis needs a proper environment that is available in the brain as neurogenesis niches (7). Although, neurogenesis does not occur sufficiently in adulthood, especially in pathological conditions (8), fortunately, today some nutritional and pharmacological stimulants are known that can enhance neurogenesis (9, 10). Neural differentiation of NSCs can be enhanced by intrinsic or extrinsic factors (11). Extrinsic factors include sexual activity (12), parental care (13), and stimulants (9, 10) such as crocin, a derivative of saffron (14).

Multipotent mesenchymal/stromal stem cells derived from various sources including adipose tissue (ADSCs) (15) can differentiate into neural lineage cells under specific conditions *in vitro *and* in vivo* (14, 16-18).

Crocin is a carotenoid that can be derived from the dried stigma of *Crocus sativus* L. (saffron) (19). This water-soluble carotenoid with the molecular formula C44H64O24 is responsible for the color of saffron. (20-22). For decades it has been used in food coloring and herbal medicine to treat infertility, enhance memory, and more (23). Previous studies have also proven that crocin may improve memory and learning (24), protect the nervous system (25), and enhance neurogenesis (26). Crocin can modulate some cellular pathways (27) including CREB/BDNF (28), Notch (29), and Wnt/β-catenin (26). These pathways, especially the CREB/BDNF and Notch pathways, are involved in neurogenesis (30-32). The Notch signaling pathway is also involved in cell differentiation (33). Blockage of Notch signaling results in the reduction of neurogenesis in adult mice (34). As well, CREB and BDNF (brain-derived neurotrophic factor) as transcription and neurotrophic factors may have some impact on neurogenesis in adult mice (35, 36). 

Since crocin has been suggested to alter Notch and CREB/BDNF signaling pathways and the possible role of these pathways in neurogenesis, we hypothesize that crocin can act as a neurogenic factor through these signaling pathways. Thus, the main aim of the current study was to investigate the impact of crocin on the neural differentiation of human adipose-derived mesenchymal stem cells in the presence of RA (Retinoic acid ), which is a key inducer for the differentiation of mesenchymal stem cells into the neural lineage. Also, it aimed to investigate the effect of crocin on the involvement of Notch and CREB/BDNF signaling pathways in the neural differentiation of ADSCs.

## Materials and Methods

 The current study was approved by the ethical review board of Kerman University of Medical Sciences, Iran (approval code IR.KMU.AH.REC.1400.113). 

***ADSCs isolation and culture***

Human adipose tissue was collected from patients referred for liposuction after obtaining written consent. ADSCs (Adipose-derived mesenchymal Stem Cells) were harvested from the adipose tissues according to the previous protocols (37). Briefly, the fat tissue was transferred to the lab under sterile conditions in PBS. Blood vessels were removed and the samples were washed with PBS containing 2% penicillin and streptomycin. The samples were diced into smaller pieces and incubated with collagenase type B for one hour. Samples were centrifuged at 300 G for 10 min and the enzyme was neutralized with 10% FBS (Fetal Bovine Serum) in PBS. Harvested cells were cultured in DMEM/F12 supplemented with 2% human umbilical cord blood serum (hUCBS ) (38) and 1% antibiotics in an incubator with 5% Co_2_ in air at 37 °C. The culture medium was changed every three days and passage 3 cells were used for the study (39). 

***Characterization of ADSCs***

*Flow cytometry*

Cells were examined for the expression of surface markers CD90 and CD105 to ensure their mesenchymal origin with flow cytometry. They were also checked for lack of expression of CD34 and CD45 markers.

*Adipogenic and osteogenic differentiation*

The proliferation and differentiation potential of ADSCs was tested by replacing the medium with the adipogenic and osteogenic medium. The adipogenic medium consisted of DMEM/F12 containing 2% hUCBS, 50 µg/ml ascorbic acid, 50 µg/ml indomethacin, and 100 nM dexamethasone. The osteogenic medium consisted of DMEM/F12 containing 2% serum, 50 µg/ml ascorbic acid, 10 mM ß-glycerol phosphate, and 100 nM dexamethasone (38). After 21 days, the differentiation medium was removed and the cells were washed with PBS. Cells were stained with oil red O to detect adipocytes and alizarin red to detect osteogenic differentiation.

*Detection of the effective dose of crocin*

Crocin was purchased with product code CP-990714 from Pouyesh Darou Sina company, Iran. According to our knowledge, no previous studies have been conducted on the use of crocin to induce neurogenesis. Therefore, the effective dose of crocin on the proliferation and differentiation of ADSCs was determined by applying different doses of crocin (from 0.1 to 2000 µg/ml) in the culture medium, and cell proliferation was evaluated by the MTT assay as follows. Fifteen thousand viable cells were seeded into each well of 96-well plates. Different doses of crocin were added to the medium. After 6 days, 1 mg/ml MTT solution was added to the wells, and after 4 hr, the MTT solution was replaced with 200 µl DMSO. One hour later, cell proliferation was evaluated with an ELISA reader (BioTek Elx800) at a wavelength of 490 nm and a reference of 630 nm.

*Cell culture and experimental design*

We prepared retinoic acid (Tretinoin) from AdooQ company with catalog number A10944 and used it as a neurogenic inducer in all groups except the control group. To investigate the possible role of the Notch signaling pathway in neurogenesis, Difluorophenyl acetamido propanamide phenylacetate (DAPT, AdooQ, A10288), an inhibitor of the Notch signaling pathway, was used (40). As well, Naphthol phosphate AS-E was purchased from Sigma Aldrich Company under catalog number 70485 which inhibits the CREB/BDNF signaling pathway (41).

Forty thousand viable ADSCs at passages 3-6 were seeded in 3 cm plates in DMEM/F12 medium with 1% hUCB serum and 1% p/s. Experiments were repeated at least twice. The most effective dose of crocin was used for further experiments in the following groups: 

A. RA group (RA): one mM RA was added to the culture medium. 

B. Crocin group (CR): 1000 µg/ml crocin and 1 mM RA were added to the culture medium. 

C. Crocin + DATP group (CR+DATP): 1000 µg/ml crocin, 10 µM DATP, and 1 mM RA (42) were added to the culture medium. 

D. Crocin + Naph group (CR+Naph): 1000 µg/ml crocin, 10 µM Napt (43), and 1mM RA were added to the culture medium. 

E. Crocin + DATP + Naph group (CR+DATP+Naph): 1000 µg/ml crocin, 10 µM DATP, 10 µM Naphthol, and 1 mM RA were added to the culture medium. 

F. Control group (Cont): received no intervention.

The treated samples along with the control were evaluated in terms of gene expression on days 7, 14, and 21, and in terms of protein expression on day 15.

*Gene expression evaluation*

Q-RTPCR was used to evaluate gene expression in Notch and CREB/BDNF pathways including HES1, Notch1, JAG1, and BDNF on days 7, 14, and 21 after treatment. First, total RNA was extracted and purified using Trizol and the extracted RNA was reverse-transcribed using the related kit (Sinagene, Tehran, Iran). q-RTPCR was performed using a MIC (Magnetic Induction Cycler) thermocycler (BMS, Australia), and the primers designed by the researchers are shown in Table 1. Note that primers were selected from the NCBI database, evaluated by Primer blast, and finally the ability to form unconventional forms was checked by Gene Runner software. Initial denaturation was carried out at 95 ºC for 15 min, followed by 40 cycles of 23-sec denaturation at 95 ºC, 30-sec annealing at 60 ºC, and 30-sec extension at 72 ºC.

*Immunofluorescence*

In the present study, a mouse monoclonal anti-CREB antibody (Santa Cruz Biotechnology, USA, sc-377154), a rabbit monoclonal anti-Doublecortin antibody (Abcam, UK, EPR10935B), a mouse monoclonal anti-NeuN antibody (Abcam, UK, 1B7), and a mouse monoclonal anti-Sox antibody (BioLegend, USA, 656109) were used. On the 15^th^ day from the start of experiments, the cells were fixed with 10% formalin and immunofluorescence was performed to evaluate the expression of the neuronal proteins CREB, DCX, SOX2, and NeuN). After fixation, washing twice with PBS, antigen retrieval with protein kinase, and blocking of non-specific antigens with non-fat milk, samples were incubated with primary antibodies against the above markers (CREB, DCX, SOX2, and NeuN) diluted in PBS for 24 hr at 4 °C. After washing twice, the samples were incubated with a secondary antibody diluted in PBS in the dark. The cells were then incubated with 0.1 μg/ml Hoechst in PBS. Finally, the samples were examined with an Olympus BX50 fluorescence microscope and images were taken with an Olympus DP72 camera to evaluate the expression of the mentioned markers.

***Statistical analysis***

Data analysis was done using GraphPad Prism 9 software. One-way ANOVA and Tukey’s *post hoc* test were used to compare quantitative variables. In addition, the Kruskal-Wallis statistical test and the Maan-Whitney *post hoc* test were used to compare the intensity of color in samples stained by immunofluorescence. *P*-value<0.05 between different groups was considered significant.

## Results

***Flow cytometry***

**Figure 1:**
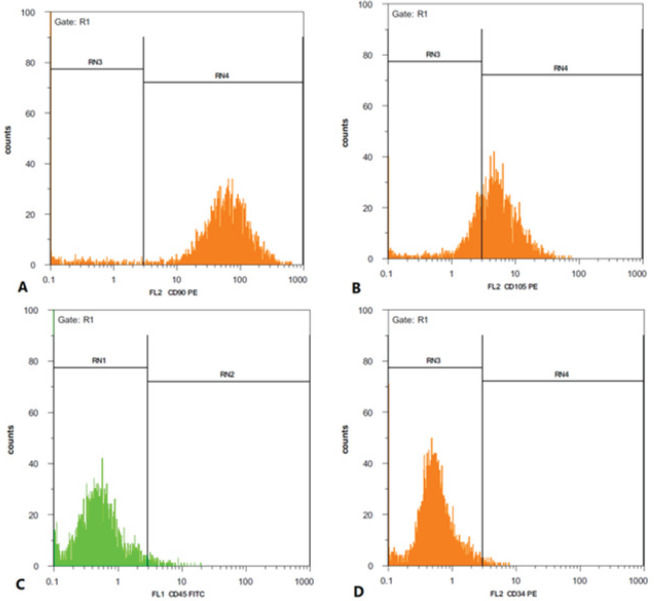
CD marker expression of harvested ADSCs. A & B: Mesenchymal markers including CD90 and CD105 were expressed by 93% and 74%. C & D: Hematopoietic markers including CD34 and CD45 were expressed 0.95% and 1.92%

Flow cytometry analysis indicated that mesenchymal markers including CD90 and CD105 were expressed by 93% and 74%, respectively (Figure 1 A & B), while hematopoietic markers including CD34 and CD45 were not expressed in these cells (0.95% and 1.92% for CD34 and CD45, respectively) (Figure 1 C & D). 

***Cell differentiation***

**Figure 2:**
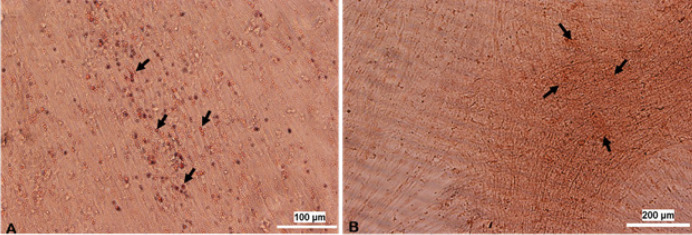
A: Adipocyte differentiation of ADSCs after 21 days. Lipid vacuoles can be seen in the cytoplasm of the treated cells (200×). B: Osteocyte differentiation of ADSCs after 21 days. Arrowheads show calcium phosphate deposits in the extracellular matrix (200×)

According to oil red O and alizarin red staining, red lipid vacuoles and calcium phosphate deposits were detectable in the cytoplasm and extracellular matrix of the treated cells after 21 days, respectively (Figure 2).

***MTT assay***

**Figure 3:**
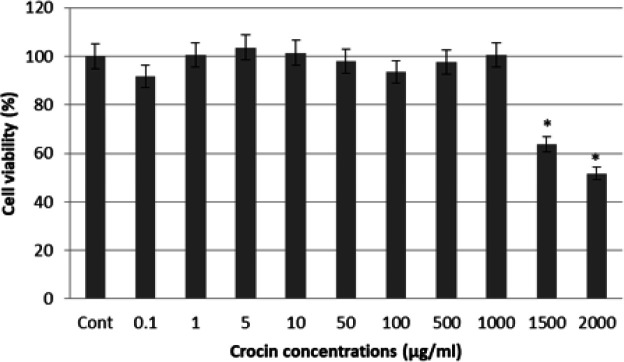
Cell viability was analyzed by MTT assay

Various doses of crocin have been reported to be proliferative in MSC cultivation (44, 45). Thus, cell viability and proliferation capacity were evaluated using an MTT assay after 6 days of treatment with different concentrations of crocin. Statistical analysis showed that after cells were exposed to 1000 μg/ml crocin no significant change in cell viability was observed compared to the control group. Meanwhile, cell viability significantly (*P*<0.05) reduced in the groups that received 1500 and 2000 µg/ml crocin. We selected the highest dose that did not affect cell viability as the optimal dose. Therefore, the highest non-toxic dose (1000 µg/ml) was used in our experiments (Figure 3). 

***Gene expression***

**Figure 4:**
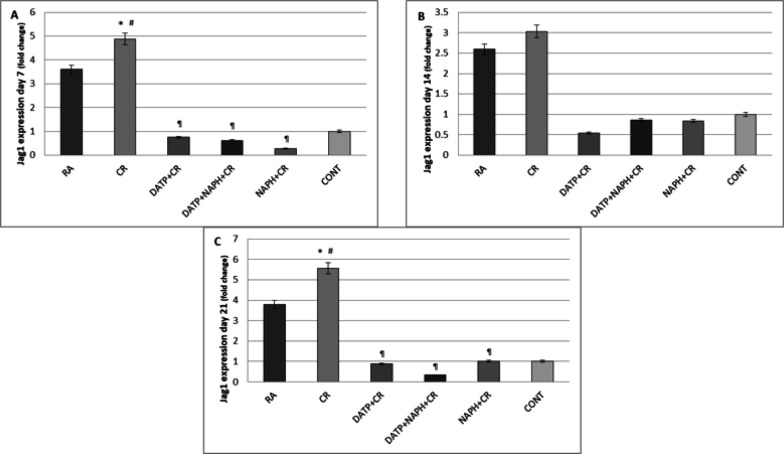
A. Jag1 expression significantly increased in CR compared to control (*) and RA (#) and decreased in inhibitor groups (¶) on day 7. B. Although the changes were detectable, they did not reach a significant level on day 14. C. On day 21 Jag1 expression significantly increased in the CR group compared to control (*) and RA (#) and decreased in the DATP and DATP+NAPH groups compared to the CR group (¶) on day 21

The data of the treated groups were normalized with the control group and demonstrated that both RA and crocin enhanced the expression of neurogenesis-related genes on different days. While the inhibitors of CREB/BDNF and Notch Pathways reduced gene expression. On day 7, Jag1 expression was significantly (*P*<0.05) increased in the crocin group compared with the RA and Control groups. The decrease in Jag1 expression in inhibitor groups was also significant (*P*<0.05) compared to the crocin group (Figure 4A). Its expression on day 14 changed non-significantly (Figure 4B), while on day 21, the expression of Jag1 increased significantly (*P*<0.05) in the CR group compared to the control and decreased significantly (*P*<0.05) in the DATP and NAPH groups compared to the CR group (Figure 4C).

**Figure 5:**
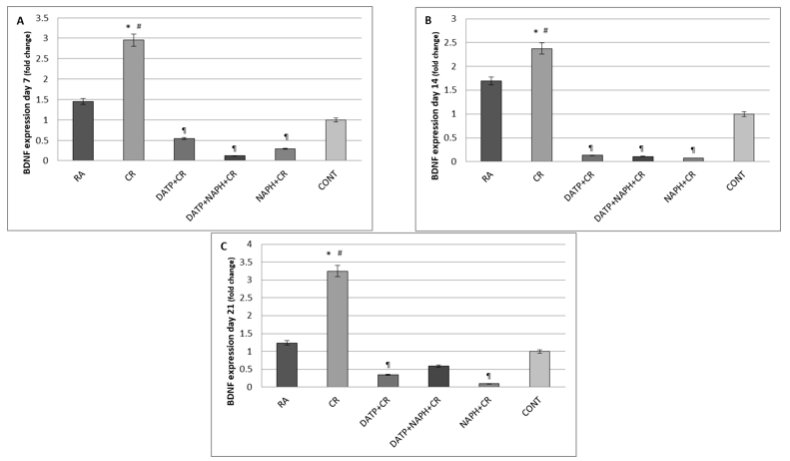
A. BDNF expression significantly increased in CR compared to control (*) and RA (#) and decreased in inhibitor groups (¶) on day 7. B. BDNF expression increased significantly in CR compared to Control (*) and RA (#) and decreased in inhibitor groups (¶) on day 14. C. BDNF expression increased significantly in CR compared to Control (*) and RA (#) and decreased in DATP and NAPH groups (¶) on day 21

BDNF expression in the CR group increased significantly (*P*<0.05) compared to the RA and control groups. Also, BDNF expression in inhibitor groups decreased significantly (*P*<0.05) compared to CR group on day 7 (Figure 5A). While, it increased significantly (*P*<0.05) on days 14 and 21 compared to the RA and control groups, and it decreased significantly (*P*<0.05) in comparison to the CR group (Figure 5B&C). 

**Figure 6:**
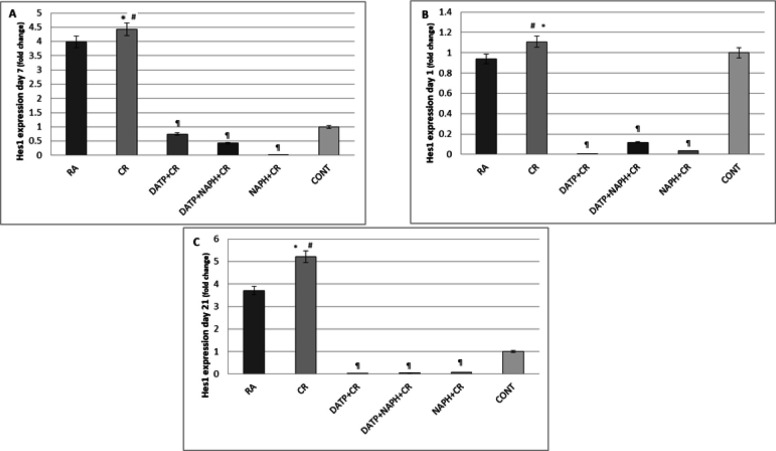
A. Hes1 expression increased significantly in CR compared to control (*) and RA (#) and decreased in inhibitor groups (¶) on day 7. B. Hes1 expression increased significantly in CR compared to Control (*) and RA (#) and decreased in inhibitor groups (¶) on day 14. C. Hes1 expression increased significantly in CR compared to control (*) and RA (#) and decreased in inhibitor groups (¶) on day 21

Expression of Hes1 in the CR group increased significantly (*P*<0.05) on days 7 and 21 compared to the RA and control groups, while decreasing significantly (*P*<0.05) in inhibitor groups compared to the CR group on day 14 (Figure 6 A&B).

**Figure 7:**
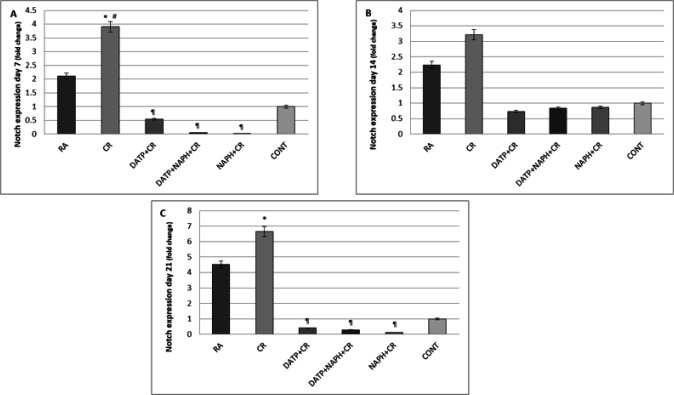
A. Notch expression increased significantly in the CR group compared to Control (*) and RA (#) and decreased in inhibitor groups (¶) on day 7. B. Although the changes were detectable, they did not reach a significant level on day 14. C. Notch expression increased significantly in CR compared to control (*) and decreased in inhibitor groups (¶) on day 21

Notch Expression on day 7 in the CR group increased significantly (*P*<0.05) compared to RA, control, and inhibitor groups (Figure 7A). The same pattern was true on day 14, except that the differences did not reach a significant level. (Figure 7B). Notch expression also increased significantly (*P*<0.05) on day 21 compared to the control group (*P*<0.05) and decreased in all inhibitor groups compared to the CR group (Figure 7C).

***Immunofluorescence***

**Figure 8:**
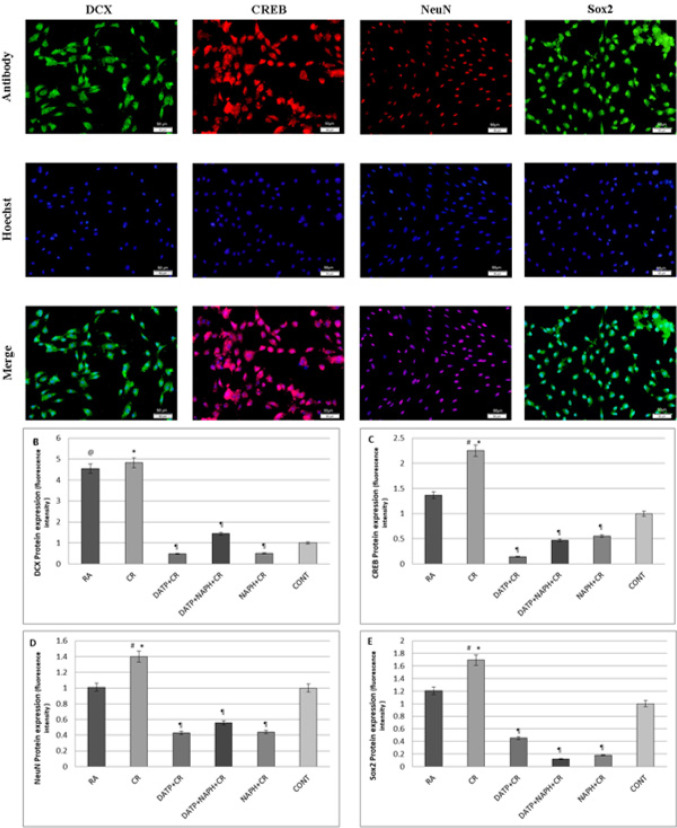
IF pictures of neuronal markers (DCX, CREB, Neun, and Sox2) expressions. Nuclei were visualized with Hoechst

Immunofluorescence results show that crocin can significantly increase the expression of all neuronal markers compared to other groups. However, the DCX expression level did not increase significantly in the CR group compared to the RA group. As shown in Figure 8B, CREB expression in the CR group increased significantly (*P*<0.05) compared to RA and Control groups, while it decreased in the inhibitor groups (*P*<0.05) compared to the CR Group. 

DCX expression increased significantly (*P*<0.05) in the RA and CR groups compared to the control and decreased in the DATP, NAPH, and DATP+NAPH groups as shown in Figure 8C. 

Another neuronal marker, NeuN significantly (*P*<0.05) expressed in the CR group more than in RA and control groups. Also, its expression was reduced significantly (*P*<0.05) in DATP, NAPH, and DATP+NAPH groups (Figure 8D).

Sox2 protein expression (Fig8E) was increased significantly (*P*<0.05) in the CR group compared to the control group. The inclusion of inhibitors (DATP, NAPH, and DATP+NAPH) in the culture medium decreased significantly (*P*<0.05) expression of sox2.

**Table 1:** The primers used in q-PCR

Gene	Sequence	
HESE1	Forward Reverse	ACTGATCACCAAGTAGCCACA GCTTTGATGACTTTCTGTGCTCA
Notch1	Forward Reverse	TACTCACGCTCTGATGCCG CGGCGTGTGAGTTGATGAG
JAG1	Forward Reverse	GCAACACCTTCAACCTCAAG AATCCCACGCCTCCACAAG
BDNF	Forward Reverse	GGAGCCAGAATCGGAACCA CCACCTTGTCCTCGGATGTT

Data (mean +/- SD) were pooled from at least 3 independent experiments in triplicate. Cell viability was significantly reduced in groups receiving 1500 and 2000 µg/ml crocin whereas no changes were obsereved in other groups. *P<0.05.

Experiments were repeated at least twice. Values were reported as Mean +/- SD. *, # and ¶ *P*<0.05.

Experiments were repeated at least twice. Values were reported as Mean +/- SD. *, # and ¶ *P*<0.05.

Experiments were repeated at least twice. Values were reported as Mean +/- SD. *, # and ¶ *P*<0.05.

Experiments were repeated at least twice. Values were reported as Mean +/- SD. *, # and ¶ *P*<0.05.

(A). Analysis of fold change in fluorescence intensity (considered as protein expression level) demonstrated that CR promoted expression of DCX (B), CREB(C), Neun(D), and Sox2 (E) compared to control (*) and RA (#). As well, RA promoted the expression of DCX compared to control (@). Meanwhile, inhibitors reduced the expression of the above markers compared to the CR group (¶). Experiments were repeated at least twice. Values were reported as Mean +/- SD. *, ¶ and @ *P*<0.05.

## Discussion

The process of neurogenesis is an effective prospect in the treatment of many neural injuries. Despite the high efficiency, this process does not proceed to the required extent in adults, especially in pathological conditions (8). This insufficiency has attracted the attention of scientists active in the field of regenerative medicine to discover new strategies to enhance neurogenesis with the help of stem cells (46). We utilized adipose tissues to harvest ADSCs, which are good candidates in regenerative medicine due to characteristics such as low immunogenicity, self-renewal, migration, and differentiation into different cell lineages, including neural lineages (47, 48). Using ADSCs, we tested different doses of crocin to find the highest dose of crocin that was safe and non-toxic, which was assessed by MTT to be 1000 µg/ml. Similar results have been reported by other researchers (49).

An *et al*. in 2020 demonstrated that crocin promotes cell proliferation and migration as well as a reduction in apoptosis in neural stem cells in a hypoglycemic deprivation/reoxygenation model. They proposed that crocin enhances cell proliferation and migration *in vitro* by boosting Notch1 expression (29). Jag1 and Hes1 are known as upstream genes of the Notch signaling pathway, and changes in their expression can influence the whole pathway as well as notch1 expression (50, 51). In line with past studies, our gene expression results reveal that simultaneous treatment of ADSCs with crocin and retinoic acid increased the expression of Notch, Jag1, and Hes1 genes (as transcription factors of the Notch signaling pathway). 

Using 1000 µg/ml crocin in ADSCs culture medium, Notch expression was significantly higher than retinoic acid except on day 14. Notably, upstream genes of the Notch pathway, Jag1 and Hes1 were expressed significantly more on this day compared to retinoic acid, which were previously reported as pioneer genes of the Notch pathway (50, 51).

Another signaling pathway that crocin affects (as previous studies have demonstrated) is the CREB/BDNF signaling pathway (52, 53). In the present study, BDNF gene expression increased in crocin-treated ADSCs compared to the RA-treated ADSCs. Also, in the presence of Naphthol as an inhibitor of the CREB/BDNF pathway, its expression was reduced significantly. Considering the fact that the treatment of ADSCs with RA was not able to significantly increase the expression of genes involved in the CREB/BDNF pathway, the higher expression of genes after crocin administration could be attributed to the enhancing effect of crocin on neurogenesis. Ahmadi *et al.* also showed an increase in the expression of BDNF and GDNF (Glial cell-derived neurotrophic factor ) after administration of crocin to epidermal neural crest stem cells *in vitro*, which suggests the ability of crocin to enhance neurogenesis (54).

Other neurogenic pathways likely to be affected by crocin include Wnt/b-catenin and Akt/GSK (55, 56). In addition, a recent study showed that 5% crocin embedded in a PVA/gelatin scaffold promoted neural differentiation of hBMSCs by increasing the expression of Nestin and Map-2 (57).

We could successfully show that crocin promotes the expression of neuronal markers DCX, BDNF, NeuN, and Sox2 expression in ADSCs, while their expression is decreased after exposure to Notch and CREB/BDNF signaling inhibitors. These findings confirm the enhancing effects of crocin on neurogenesis as revealed by the expression of particular genes involved in CREB/BDNF and Notch pathways. Previously, it has been shown that crocin administration in adolescent mice can enhance DCX expression (14). In agreement with our results, Vahdati Hassani *et al.* showed that BDNF expression as well as p-CREB, CREB, and VEGF protein expression was promoted after crocin administration in the hippocampus of rats (58). A study in 2022 demonstrated that the antidepressant effects of crocin were associated with hippocampal neurogenesis in mice, which was confirmed by the expression of DCX and NeuN in the hippocampus of mice (59). The results of this study support our previous research on the possible effect of crocin in promoting the expression of neuronal markers and enhancing neurogenesis.

## Conclusion

Our results indicated that crocin enhances *in vitro* neurogenesis in human adipose-derived mesenchymal stem cells by affecting gene expressions as well as by increasing the expression of neural proteins through Notch and CREB/BDNF signaling pathways. Further *in vivo* and clinical studies are required to understand other signaling pathways involved in the neurogenesis following crocin administration. Whether crocin in the absence of neural inducers can affect neurogenesis *in vitro* is beyond the scope of our study and needs further investigations. 

## Authors’ Contributions

All authors conceived and designed the research. S V and E M performed experiments and collected data. S V and S N analyzed data. S N supervised, directed, and managed the study. S V, S N, V M, and M B wrote the manuscript. All authors read and approved the final version to be published.

## Conflicts of Interest

The authors declare no conflicts of interest.
